# Effects of the Incredible Years parenting program on sibling conduct problems: A latent transition analysis

**DOI:** 10.1002/jcv2.70006

**Published:** 2025-04-16

**Authors:** Elise Sellars, Lucy Bowes, Bonamy R. Oliver, Frances Gardner, Judy Hutchings, Sinéad McGilloway, G. J. Melendez‐Torres, Patty Leijten

**Affiliations:** ^1^ Department of Experimental Psychology University of Oxford Oxford UK; ^2^ IOE‐ Psychology & Human Development UCL Institute of Education University College London London UK; ^3^ Department of Social Policy and Intervention University of Oxford Oxford UK; ^4^ Centre for Evidence Based Early Intervention Bangor University Bangor UK; ^5^ Centre for Mental Health and Community Research Department of Psychology Maynooth University Maynooth Ireland; ^6^ Faculty of Health and Life Sciences University of Exeter Exeter UK; ^7^ Research Institute of Child Development and Education University of Amsterdam Amsterdam The Netherlands

**Keywords:** conduct problems, Incredible Years, latent transition analysis, parenting program, siblings

## Abstract

**Background:**

Behavioral parenting programs are a primary strategy used to reduce children's conduct problems. Although behavior problems in siblings may co‐occur, behavioral parenting program trials typically report outcomes for one child per family (the index child), with potential program effects on any non‐targeted sibling largely neglected. This study examined co‐occurring patterns of index child and non‐targeted sibling conduct problems, and how parental participation in the Incredible Years (IY) program changes these patterns.

**Methods:**

We used individual participant data pooled across three randomized trials of the IY parenting program in England, Wales, and Ireland, with data for the index child and one non‐targeted sibling (*N* = 240 families, 480 children; index child: *M* age = 4.73, *SD* = 1.44, range 2–9 years, 62% male; non‐targeted sibling: *M* age = 5.94 years, *SD* = 3.15, range 6 months−15 years, 49% male). We used latent transition analysis to identify latent classes at both baseline and posttest based on families' combinations of index child and non‐targeted sibling conduct problems.

**Results:**

We identified two classes with distinct patterns of co‐occurring sibling dyad conduct problems: one with moderate clinical levels of index child conduct problems and non‐clinical levels for the non‐targeted sibling; and one with severe clinical levels for both children. In terms of the effects of IY, most intervention families maintained their patterns of sibling dyad conduct problems, but with lower levels across classes. Most intervention families reported improvements predominantly for the index child. However, a minority of families with severe baseline levels of conduct problems in both children moved to a class with non‐clinical levels for both children.

**Conclusions:**

For most families, IY had limited effects on non‐targeted sibling disruptive behavior. However, IY may reduce co‐occurring sibling conduct problems for a small number of families with initially severe levels in both children.

**Key points**

- What's known: Behavioral parenting programs, such as Incredible Years (IY), are widely used to help reduce children's conduct problems. Program trials typically report outcomes for one child per family (index child), neglecting potential effects on non‐targeted siblings.
- What's new: This study examined co‐occurring patterns of conduct problems in the index child and their sibling, using data from three randomized trials of IY conducted in three countries. Findings indicate that IY primarily reduces conduct problems in the index child.
- What's relevant: Of relevance for clinical practice, it is not guaranteed that a parenting intervention will benefit the non‐targeted sibling in families where two children have severe conduct problems. Future research should consider how to support these families to ensure that intervention benefits extend beyond the index child.

## INTRODUCTION

Although siblings can be very different, behavior problems often co‐occur in siblings (Defoe et al., 2013). This is especially the case in families referred to treatment for one child's conduct problems (Brestan et al., 1997; Smorti et al., 2021). Yet, intervention effects on children's conduct problems are typically reported for just one child per family (i.e., the ‘index’ child, usually defined as the child who has been referred, or with the most severe conduct problems in the family). This raises questions about possible intervention effects on the wider family system: for example, does only the index child benefit, or do siblings benefit too? Does the relative level of conduct problems in siblings within the same family make a difference? These are crucial questions given that most families have multiple children. If parenting programs benefit more than one child per family, their current public health impact might be underestimated. Conversely, if the conduct problems of siblings do not reduce, existing programs may need to be adapted to ensure their benefits extend beyond the index child.

Several mechanisms may explain how siblings mutually develop patterns of conduct problems. First, genetic factors are likely to contribute, as biological (non‐identical) siblings are 50% genetically similar, and conduct problems are heritable (Ferguson, 2010). Second, siblings may share experiences of coercive interactions with parents, especially because sibling conflict is a known stressor for parents (Tucker & Kazura, 2013), and parental stress increases the likelihood of parents implementing harsh parenting practices (Dănilă et al., 2024). Third, siblings may directly influence each other's conduct problems, by virtue of their everyday interactions. For example, siblings can ‘train’ conduct problems in each other, learning that behaving in an increasingly aggressive manner toward their sibling results in ‘winning’ an argument with them (Patterson, 1984). Fourth, siblings may collude to form alliances which promote conduct problems and undermine parental authority (Bullock & Dishion, 2002). Sibling collusion is predicated on a pattern of mutual positive reinforcement of ‘deviant talk’‐ for example, a sibling providing laughter/interest when another sibling breaks a family rule, reinforcing child conduct problems.

Despite similarities between siblings, they may also differ in their level of conduct problems (Oliver & Pike, 2018). Behavioral genetics research highlights the influence of the non‐shared environment on these differences (Plomin & Daniels, 2011). For example, negative parental differential treatment, whereby parents direct more hostility toward one child compared to their sibling, is associated with sibling behavior differences. Meta‐analytic evidence from 13 samples of over 7000 sibling pairs, suggests that the sibling who received less parental warmth and more hostility, relative to their sibling, also showed higher levels of externalizing problems (Eradus et al., 2024). Negative differential treatment may be particularly pertinent in the context of children with high levels of conduct problems, who may be subject to harsher parental discipline levels than their sibling, thereby reinforcing the cycle of coercive parent‐child interactions, and further entrenching differential treatment. Non‐shared environmental effects may also reside in experiences outside of the home, such as differences between siblings in their experiences with peers (Plomin & Daniels, 2011). Additionally, siblings may differ in their levels of conduct problems if, when one sibling has high levels of conduct problems, the other sibling assumes a caring role to help ensure the smooth functioning of the family system. One child may also observe the negative consequences of their sibling's conduct problems (e.g., harsh parental discipline) and choose to refrain from similar behaviors (Daniel et al., 2018).

The effectiveness of behavioral parenting programs in reducing children's conduct problems has been demonstrated in hundreds of randomized trials (Beelmann et al., 2023). For example, there is robust evidence that the well‐known Incredible Years (IY) parenting program can effectively reduce child conduct problems (Leijten et al., 2018), and is recommended, therefore, by several influential bodies (e.g., National Institute for Health and Care Excellence (UK)) for the prevention and treatment of conduct problems. As with other behavioral parenting programs, IY teaches parents to engage more positively with their child(ren) and reduce patterns of coercive interaction in which they and their child unwittingly reinforce aversive behavior in each other. Coercion can create cycles of interactions that become increasingly difficult to manage, often leading to the development of child conduct problems (Patterson, 1982).

Family systems theory, which emphasizes the interdependence of relationships within a family (Carr, 2016), offers a useful conceptual framework to understand the ways in which parenting programs that target the index child may also help to reduce the conduct problems of a non‐targeted sibling. For example, parents' increased ability to avoid or break coercive interactions with one child, may allow them to also do so with their other children (Weeland et al., 2021). In addition, given the bidirectional relation between sibling relationship quality and individual child behavior (Pike & Oliver, 2017), reduced index child conduct problems may benefit the sibling relationship, and subsequent behavior of both the index child and their sibling. Last, because parent‐child conflict is positively associated with sibling conflict (McHale et al., 2024), a reduction in coercive parent‐index child interactions may also reduce problem behavior in the non‐targeted sibling. Indeed, three small IY trials which included non‐targeted siblings reported either immediate (Gardner et al., 2006; Hutchings et al., 2007) or delayed (McGilloway et al., 2014) intervention effects in this group. Similar findings were reported in a trial of Parent‐Child Interaction Therapy (Brestan et al., 1997).

It is possible that in some families, the conduct problems of the non‐targeted sibling worsen following parental participation in a parenting program. Indeed, differential treatment by parents is a known risk factor for the development of conduct problems in the non‐favored sibling. Consequently, if changes in parenting behavior (such as praising desirable child behavior) are predominantly applied within the parent‐index child relationship, the non‐targeted sibling may perceive that the index child receives more attention and praise for their improved behavior. This, in turn, may lead to feelings of exclusion and sibling rivalry, and subsequent increases in non‐targeted sibling conduct problems (Weeland et al., 2021).

The few IY trials that reported non‐targeted sibling outcomes used a variable‐centered approach, separately analyzing intervention main effects for the index child and their sibling. This approach overlooks the interlinked nature of sibling conduct problems and focuses on population‐level relationships between variables. As such, it remains unclear what co‐occurring patterns of sibling dyad conduct problems might exist, and whether there is heterogeneity in sibling dyad responses to IY.

Latent transition analysis (LTA), a person‐centered approach (i.e., focusing on how variables relate within families), is well‐suited for addressing these research gaps. LTA identifies subgroups (latent classes) of families distinct in their co‐occurring patterns of indicator variables (e.g., index child and non‐targeted sibling conduct problem scores). It then models how families transition between these subgroups from baseline to posttest in the presence of a covariate (e.g., intervention status), revealing variation in how families respond to the intervention. For example, one baseline class might consist of families where both siblings have severe conduct problems, with most transitioning to a posttest group where both exhibit non‐clinical levels. However, a minority may transition to a group where only the index child's conduct problems improve. Such nuanced patterns, which would be obscured in variable‐centered approaches, are crucial for understanding heterogeneity in families' responses to IY and potentially tailoring the intervention accordingly. Person‐centered approaches have been shown to effectively identify and explain heterogeneity in family responses to parenting programs (Pelham et al., 2017; Van Aar et al., 2019).

Identifying differences between families in changes to patterns of the behavior of two children requires a sufficiently large sample size. Synthesizing individual family level data across trials into one integrated data set achieves this, as variance both between and within trials can be used to estimate differential program benefits, increasing statistical power and generalizability relative to data from an individual trial (Curran & Hussong, 2009).

### The present study

The aims of this study were to: (1) examine patterns of co‐occurring patterns of index child and sibling conduct problems; and (2) ascertain the influence of parental participation in the IY parenting program on change in these co‐occurring patterns. We expected differences between families in the co‐occurrence of children's conduct problems, and variation in how sibling dyads respond to the intervention.

## METHODS

### Design and procedure

We used a pre‐existing set of individual family level data from 15 randomized trials on the effects of the IY parenting program for children aged 0–12 years (Leijten et al., 2018; Sellars et al., 2024). Trials were included only from European countries to help ensure relative homogeneity in the usual services that children received across trials, thus supporting the comparability of the pooled data. All trials were conducted by researchers independent of the program developer. We preregistered our study procedures on OSF .

We included data from trials in which both of the following criteria were met: (a) families were recruited based on the conduct problems of one child (the index child) per family; and (b) the trial collected baseline and posttest (defined as first measurement point after intervention termination) conduct problem data for the index child and a non‐targeted sibling. Three trials were eligible for inclusion (Table 1).

**TABLE 1:** Overview of trial characteristics.

Trial	Lead author (year)	Country	Setting	Families	Index child age (mean)	Sibling age (mean)	Index child baseline conduct problems (mean)	Sibling baseline conduct problems (mean)	% Low income	% Ethnic minority
1	Gardner et al. (2006)	England	Community services	40	2–9 (6.35)	2–13 (5.70)	55–199 (158.74)	58–191 (121.00)	62	0
2	Hutchings et al. (2007)	Wales	Community services	106	3–4 (3.89)	0–15 (5.23)	75–218 (145.57)	53–225 (126.70)	80	1
3	McGilloway et al. (2012)	Ireland	Community services	94	2–7 (5.00)	2–14 (6.67)	87–235 (159.64)	46–222 (113.51)	44	5

*Note*: Numbers reported in this study differ from those reported in individual trials, due to its inclusion criteria (and because some families might not have more than one child). Possible range of conduct problem scores (Eyberg Child Behavior Inventory; ECBI) is 36–252. An average community sample score is 109, scores >131 are considered to indicate clinical levels of conduct problems that is, above the 80th percentile (Burns & Patterson, 2001).

Within these trials, 64% of families were included in the current study; the remaining 36% were excluded because they did not have sibling data for both baseline and posttest assessments. In comparison to included families, excluded families had siblings that were on average younger, and parents were more likely to be a teen or lone parent than families included in the current study (Table S1).

All trials recruited families from community service settings, based on high levels of conduct problems in the index child. When families had more than two children, trial researchers selected which non‐targeted sibling to collect data from using the following criteria: Trial #1 (Gardner et al., 2006) collected conduct problem data for the sibling whom parents considered to be ‘the next most difficult’; trials #2 (Hutchings et al., 2007) and #3 (McGilloway et al., 2012) collected data from the sibling who was closest in age to the index child. Each trial received ethical approval from its respective internal Ethical Review Board, and the protocol for the original pooling study protocol was reviewed by the Departmental Research Ethics Committee of the Department of Social Policy and Intervention, University of Oxford.

### Participants

The total sample across the three trials included 240 families (175 intervention and 65 control; two trials used a 2:1 allocation). Index children were aged 2–9 years (*M* = 4.73 years, *SD* = 1.44) and predominantly male (62%). Non‐targeted siblings were aged 6 months −15 years (*M* = 5.94 years, *SD* = 3.15) and 49% male. Because of this wide age range, we conducted sensitivity analyses to see if findings depended on sibling age (see ‘Post‐hoc analyses’ results section). The non‐targeted sibling was older than the target child in 60% of dyads. The mean age gap between siblings was 2.14 years (*SD* = 2.15). Same gender dyads accounted for 48% of the sample (of which 63% were brother‐brother dyads). At baseline, 78% of index children and 34% of the non‐targeted siblings scored above the 80^th^ percentile on the ECBI ‐ see Burns and Patterson (2001) for norm scores.

Parents were aged 19–55 years (*M* = 32.50 years, *SD* = 6.18). The socioeconomic status of families was diverse (63% low income; 51% low educational level; 52% no employed parent in the household). The sample was not diverse in terms of including families from an ethnic minority (2.6%), reflecting the localities in which the trials were conducted. We used data from one parent (98% mothers) because most trials included data from one parent only. There were no statistically significant differences between conditions at baseline (Table S2).

### Intervention and control condition

The IY parenting program aims to reduce children's conduct problems by teaching parenting techniques to build warm and nurturing parent‐child relationships, encourage positive child behavior, and discourage negative child behavior (Webster‐Stratton, 2015). The program is delivered solely to parents; children do not attend the sessions. Two group leaders and parents work together on how different strategies can best be used with children of different ages (e.g., ‘time‐out’ for younger children and removal of privileges for older children). Techniques are taught using practice‐based methods, including discussions of videos modeling parenting strategies and role‐play exercises. A key aspect of the program is its use of a collaborative delivery style; content is first presented to all members of the group, then leaders work with individual parents to help them identify key principles from the content that can be applied to their specific parenting situations and goals. Parenting goals may or may not include the behavior of children in the home other than the index child. While the program does not contain specific materials which address the generalization of skills to all children in the family, certain elements, such as the ‘praise’ section, encourage parents to extend praise to others in the family, such as partners and siblings. The extent to which parents are encouraged to apply the strategies learned in the program with all children in the family depends on the group leaders, group dynamics, and the goals of individual parents. The number of sessions ranged from 12 (trial #2 and #3) to 14 (trial #1). Of the parents who attended at least one session (87%), parents attended on average 72% of sessions. All trials used waitlist controls.

### Measures

#### Conduct problems in the index child and their sibling

All trials measured index child and sibling conduct problems at baseline and posttest using the parent‐reported Eyberg Child Behavior Inventory Intensity Scale (ECBI; Robinson et al., 1980), a well‐established measure of child disruptive behavior. Possible total scores range from 36–252 with higher scores indicating greater levels of disruptive behavior. The internal consistency for both index child and sibling measures were high (α 0.93–0.95).

#### Analytic strategy

We used LTA as a person‐centered approach to describe and classify families into classes (i.e., subgroups) distinct in their co‐occurring patterns of index child and non‐targeted sibling conduct problem scores. We described classes separately for baseline and posttest data respectively. LTA could then be used to estimate families' transitions between classes that exist at either time point, and how these might vary according to intervention status.

Analyses were preregistered [ OSF link], and conducted in Mplus version 8.10 (Muthén & Muthén, 2018). First, we estimated sequential latent class solutions (2–4 classes) on baseline and posttest index child and sibling conduct problem data simultaneously, to determine the optimal number of latent classes in our data. We used clustered standard errors to account for data coming from three different trials. We fixed indicator variances to be equal across classes and waves to preserve interpretability between classes and waves. We did not require within‐class indicator means to be equal across waves, as examination of our class solutions indicated sibling dyad conduct problems decreased across all classes at posttest.

We chose the optimal latent class solution using a combination of fit indices (Table 2) and theoretically informed judgment. We focused mainly on the degree to which improvement in model fit slowed down when testing models with more classes. Smaller AIC, BIC, and aBIC values suggest improved model fit, whereas a larger entropy value indicates more accurate and precise classification of families. The bootstrap likelihood ratio test was not used as it is not available for clustered data.

**TABLE 2:** Model fit of the latent transition analysis (LTA).

	AIC	BIC	aBIC	Baseline entropy	Posttest entropy
2	9033	9096	9039	0.77	0.78
3	9038	9149	9047	0.87	0.83
4	8971	9145	8986	0.86	0.83

*Note*: Model fit without the covariate.

Abbreviations: aBIC, sample size adjusted Bayesian information criterion; AIC, Akaike information criterion, BIC, Bayesian information criterion.

We examined how intervention status predicted movement between classes by testing a one‐step interaction model between class membership at baseline and intervention status in predicting class membership at posttest. We directly estimated probabilities of movement between classes for intervention and control families using a probability parametrization. We used full information maximum likelihood (FIML) to account for missing data.

## RESULTS

### Descriptive results

The correlation between index child and sibling ECBI total scores at baseline was low (*r* = 0.07) and statistically non‐significant; at posttest, the correlation was moderate and significant (*r* = 0.32, *p* < 0.001). The intervention reduced index child conduct problems (β −0.53, 95% CI −0.84 to −0.38, *p* < 0.001). There was a smaller, non‐significant main effect of the intervention on sibling conduct problems (β −0.10, 95% CI −0.37 to 0.17, *p* = 0.344).

### Subgroups of co‐occurring patterns of sibling dyad conduct problems

We tested between two and four latent class solutions (Table 2). Increasing model complexity did not result in large improvements in model fit. Although a four‐class solution indicated some minor improvements in model fit, it produced a class with just two families, suggesting overextraction of classes. Furthermore, BIC generated the best value at two classes, which has been proposed as the best metric for model comparisons (Nylund et al., 2007). With these factors in mind, we chose a two‐class solution.

The largest group at baseline (*N* = 192, 80% families; Table 3) included families where index child conduct problems were of a clinical level (*M* = 150, 131 is considered the clinical cut‐off (Burns & Patterson, 2001)) and non‐targeted sibling conduct problems were not (*M* = 108). The smaller baseline group (*N* = 48, 20% of families) included families where conduct problems were severe for both children (*M*_*target child*_ = 166; *M*_*sibling*_ = 171).

**TABLE 3:** Baseline probability for each class.

Variable	Class 1	Class 2
Baseline probability	80%	20%
Index child conduct problems		
Baseline *M*	150.12	165.95
Posttest *M*	123.11	148.04
*S* ^2^ ^a^	1073.84	1073.84
Sibling conduct problems		
Baseline *M*	107.66	171.37
Posttest *M*	98.24	167.98
*S* ^2^ ^a^	774.94	774.94

*Note*: For model with the covariate.

Abbreviations: *M* = mean, *S =* variance.

^a^ Variance was held constant.

**FIGURE 1:**
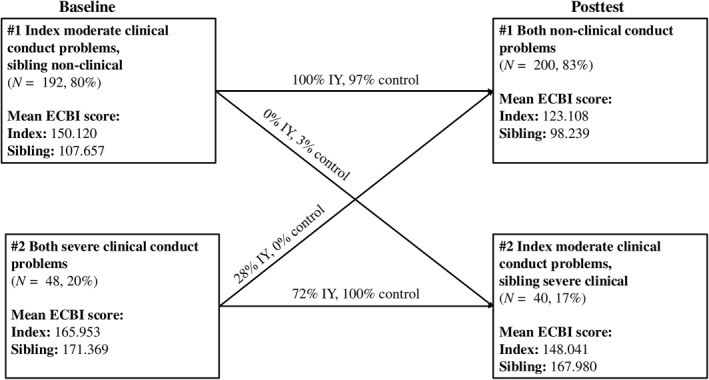
Intervention effects on families' transitions between classes. Class labels refer to the mean levels of index child and non‐targeted sibling conduct problems in that class.

Class distributions were largely similar at posttest. Similar patterns were observed at posttest, but with lower levels of conduct problems across classes (Figure 1).

### Effects of the Incredible Years program on change in co‐occurring patterns of sibling conduct problems

All intervention families in baseline class #1 remained in a class with more severe index child than sibling conduct problems at posttest, although mean index child conduct problems were no longer at a clinical level (Figure 1).

Intervention families in class #2 had a 72% likelihood of remaining in a posttest class with a similar pattern of sibling dyad conduct problems at posttest, but with mean index conduct problems now at moderate clinical levels. However, families in this class also had a 28% likelihood of transitioning to a class defined by a different pattern of sibling dyad conduct problems, with non‐clinical conduct problem levels for both siblings.

### Post‐hoc analyses [not pre‐registered]

The age range of non‐targeted siblings in our study was 6 months−15 years (*M* = 5.94 years, *SD* = 3.15). This raised several potential issues: the ECBI is not designed for children under the age of two, and the IY program is not designed for parents with children over the age of 12. However, descriptive statistics for the small number of siblings under the age of two (*n* = 18; 8%; just one child < one year old [6 months]) and over the age of 12 (*n* = 6; 3%) did not differ from the main sample. Furthermore, sensitivity analyses excluding these families from the study sample did not change the main effects (Table S3). Therefore, to maximize our study sample, we retained these families in our LTA models.

We also explored sibling dyad and family characteristics across different transition patterns (Tables S4 and S5, respectively). Child characteristics, such as sibling dyad age gap and gender, were similar across the different transition patterns (Table S4), as were family characteristics, including the age of the main carer and income level (Table S5). However, families with the greatest reductions in conduct problems across the sibling dyad (group 5, Table S5) attended slightly more IY sessions (an average of 10 sessions) than families following different transition patterns (seven or eight sessions).

## DISCUSSION

This study examined how sibling dyads are distinct in their co‐occurring patterns of conduct problems and how these patterns change in response to parental participation in the IY program. We identified one subgroup at baseline characterized by moderate clinical levels of index child conduct problems and non‐clinical levels of non‐targeted child conduct problems (80% of families), and one characterized by severe levels of conduct problems for both siblings (20% of families). Most intervention families maintained their patterns of co‐occurring sibling dyad conduct problems at posttest, but with lower levels of sibling dyad conduct problems across classes. Most intervention families saw improvements in conduct problems predominantly for the index child. However, consistent with our hypotheses, sibling dyads responded to the intervention in different ways; among intervention families with the most severe clinical conduct problems in the sibling dyad at baseline, a small minority moved to a class characterized by non‐clinical levels of conduct problems for both siblings, while most stayed in the same class.

Our finding that conduct problems reduced mainly in index children may be because in most families, the index child was perceived by parents as having the most severe conduct problems, so therefore parents may have prioritized strategies to reduce the index child's conduct problems. Additionally, the generally low levels of baseline conduct problems in the non‐targeted siblings might mean that for many children, their scores would not reduce further.

We also found that some families with severe conduct problems in both children benefited considerably from the intervention, transitioning to a posttest class characterized by non‐clinical levels of conduct problems for *both* children. This is in line with previous research which has repeatedly found that the strongest parenting intervention benefits are observed for families where there is more scope for behavior change to occur (Van Aar et al., 2019). However, it is important to note that most families with severe conduct problems in both children (72%), did not follow this transition pattern, remaining in a posttest class characterized by moderate or severe clinical levels of conduct problems in the index child and non‐targeted sibling respectively. This suggests that the main effects typically seen in such interventions may mask the fact that many families do not benefit from the intervention (relative to the control condition, where most families also report improvements), while a small group of families benefit greatly.

We propose several explanations as to why only some families with severe clinical levels of conduct problems in both children benefit from the intervention, in terms of reductions in conduct problems for both children. First, it may be that in families where there was no reduction in non‐targeted sibling conduct problems, parents predominantly applied the skills and strategies acquired during the IY program to address the behavior of the index child. Therefore, the non‐targeted sibling may not have received enough of the newly acquired parenting skills to elicit a change in their level of conduct problems at this timepoint. In contrast, families where both children benefitted from the intervention may have applied their newly acquired parenting skills equally to both children. Notably, families where both children benefitted from the program attended, on average, slightly more intervention sessions. This may have helped parents generalize their newly acquired parenting skills to both children. Unfortunately, we were unable to explore this in our study, due to collection of parenting behavior data for the index child only. It is also not clear to what extent group leaders supported parents in generalizing new behavior patterns to siblings. As the program does not contain formal materials on this, leader encouragement is likely to be crucial. Indeed, previous research demonstrates that leader skills play a key role in fostering parent behavior change in IY (Eames et al., 2010). To enhance our understanding of the importance of leader support in generalization of skills, future trials could consider monitoring this as part of program implementation fidelity. Relatedly, it is important to also consider how to support parents when there is a larger age gap between siblings, as this may make it more challenging to generalize skills practiced with the index child.

This is the first study to examine co‐occurring patterns of sibling conduct problems and how parental participation in a parenting program might change these patterns, providing a novel contribution to our understanding of the effects parenting programs on family members beyond the index child. This helps to inform the potential public health impact of such programs, indicating that although in most families, non‐targeted sibling conduct problems do not reduce to the same extent as those of the index child, IY influences the behavior of non‐targeted siblings for a minority of families with severe problems at baseline. This is in line with earlier findings that IY results in co‐occurring improvements in index child conduct problems and maternal depressive symptoms in a small subgroup of families with the most severe problems (Leijten et al., 2019).

Our finding that 20% of families had severe levels of conduct problems in both children at baseline, despite being recruited to trials due to conduct problems in the index child, also has implications for practice. For example, it is important to consider how to effectively support these families to ensure that intervention benefits generalize beyond the index child. This is particularly pertinent, considering our finding that almost three‐quarters of families with severe clinical conduct problems in both children maintained clinical levels of these problems.

The use of individual family‐level data synthesized across trials helped maximize transparency and reduce selective outcome reporting, by including all available data in the pooled dataset. Synthesizing data across several trials also provided us with a sufficiently large sample size to utilise a person‐centered approach to our analyses, enabling us to examine subgroups of index child and non‐targeted siblings with different patterns of co‐occurring conduct problems. This is a key strength of this study, as had we examined only the overall effects of IY on index child and non‐targeted sibling conduct problems, we would have missed the small subgroup of intervention families who went from severe levels of conduct problems for both children to non‐clinical levels of conduct problems for both children. To extend this finding, future research could also assess the magnitude of changes in sibling conduct problems, which was precluded by sample size in the current study.

This study also has limitations. Although we integrated data from several trials, only a small number of trials included sibling data. This reflects the general tendency in the behavioral parenting program literature to focus on only one child per family, rather than program effects on the wider family system (Weeland et al., 2021). It may also be beneficial to consider data from more than two children in a family; however, to our knowledge, only one parenting program study has included data for more than two children per family (Menting et al., 2014). Additionally, 36% of families were excluded from our study because they did not have sibling data for both baseline and posttest assessments. The pooled dataset does not contain information on whether this missing data resulted families having only one child, or from two‐child families who did not complete the sibling ECBI. Excluded families were more likely to have caregivers who were teen or lone parents ‐ both potential risk factors for child conduct problems. Consequently, if these excluded families had two children, the prevalence of families with severe conduct problems in both children at baseline (20% of our sample) may have been underestimated.

There are also limitations related to the study measures. Conduct problems in sibling dyads were assessed solely through parent report, as observational ratings of non‐targeted sibling behavior were not collected in the trials in comprising our pooled dataset. However, a recent meta‐analysis on the effects of parenting programs on child disruptive behavior found no difference between parent‐rated and observed effects (Beelmann et al., 2023). Nonetheless, when parents rate the disruptive behavior of more than one child in their family, this may be subject to contrast effects, whereby parents perceive the disruptive behavior of siblings as more different than it is. Therefore, it may be beneficial to corroborate our findings with observational measures of sibling dyad disruptive behavior. Finally, while our analytic approach described variations in family responses to IY, our sample may not have been sufficiently large to reveal additional response patterns. The use of only two indicators (index child and sibling ECBI) may also play a role. Larger samples with more diverse indicators of children's behavior may reveal further response patterns.

To conclude, we identified two subgroups of families with distinct patterns of sibling conduct problems at baseline. Most families had moderate clinical levels of index child conduct problems and non‐clinical levels for the non‐targeted sibling, while a minority had severe clinical levels for both children. IY primarily reduced conduct problems in the index child. This suggests that, although some families have two children with severe conduct problems, only a small number of these families benefit from IY in terms of *both* children having non‐clinical levels of conduct problems following the intervention. Consequently, it is not guaranteed that the non‐targeted sibling will also benefit from IY. Our findings therefore represent an important addition to the literature on parenting programs and highlight the need to better understand how to support families to ensure that, where applicable, the benefits of such programs extend to more than one child per family.

## AUTHOR CONTRIBUTIONS

**Elise Sellars:** Conceptualization; formal analysis; writing – original draft; writing – review & editing. **Lucy Bowes:** Conceptualization; supervision; writing – review & editing. **Bonamy R. Oliver:** Conceptualization; supervision; writing – review & editing. **Frances Gardner:** Data curation; writing – review & editing. **Judy Hutchings:** Data curation; writing – review & editing. **Sinéad McGilloway:** Data curation; writing – review & editing. **G. J. Melendez–Torres:** Formal analysis; writing – review & editing. **Patty Leijten:** Conceptualization; supervision; writing – review & editing.

## CONFLICT OF INTEREST STATEMENT

The authors declare no conflicts of interest.

## ETHICAL CONSIDERATIONS

Ethical approval was not applicable for this study, as it is a secondary data analysis of pre‐existing pooled trial data. Each trial received ethical approval from its respective internal Ethical Review Board, and the protocol for the original pooling study protocol was reviewed by the Departmental Research Ethics Committee of the Department of Social Policy and Intervention, University of Oxford.

## Supporting information

Tables S1–S5

## Data Availability

The data that support the findings of this study are available from the corresponding author upon reasonable request.

## References

[jcv270006-bib-0001] Beelmann, A. , Arnold, L. S. , & Hercher, J. (2023). Parent training programs for preventing and treating antisocial behavior in children and adolescents: A comprehensive meta‐analysis of international studies. Aggression and Violent Behavior, 68, 101798. 10.1016/j.avb.2022.101798

[jcv270006-bib-0002] Brestan, E. V. , Eyberg, S. M. , Boggs, S. R. , & Algina, J. (1997). Parent‐child interaction therapy: Parents’ perceptions of untreated siblings. Child & Family Behavior Therapy, 19(3), 13–28. 10.1300/J019v19n03_02

[jcv270006-bib-0003] Bullock, B. M. , & Dishion, T. J. (2002). Sibling collusion and problem behavior in early adolescence: Toward a process model for family mutuality. Journal of Abnormal Child Psychology, 30(2), 143–153. 10.1023/A:1014753232153 12002395

[jcv270006-bib-0004] Burns, G. L. , & Patterson, D. R. (2001). Normative data on the Eyberg child behavior inventory and Sutter‐Eyberg student behavior inventory: Parent and teacher rating scales of disruptive behavior problems in children and adolescents. Child & Family Behavior Therapy, 23(1), 15–28. 10.1300/J019v23n01_02

[jcv270006-bib-0005] Carr, A. (2016). The evolution of systems theory. In T. L. Sexton & J. Lebow (Eds.), Handbook of family therapy: The science and practice of working with families and couples (pp. 13–29). Routledge.

[jcv270006-bib-0006] Curran, P. J. , & Hussong, A. M. (2009). Integrative data analysis: The simultaneous analysis of multiple data sets. Psychological Methods, 14(2), 81–100. 10.1037/a0015914 19485623 PMC2777640

[jcv270006-bib-0007] Daniel, E. , Plamondon, A. , & Jenkins, J. M. (2018). An examination of the sibling training hypothesis for disruptive behavior in early childhood. Child Development, 89(1), 235–247. 10.1111/cdev.12754 28195432

[jcv270006-bib-0008] Dănilă, I. , Balazsi, R. , Tăut, D. , Băban, A. , Foran, H. M. , Heinrich, N. , Lachman, J. M. , & Hutchings, J. (2024). Linking child adjustment difficulties with mother’s maladaptive parental behavior: The mediating roles of parental cognitions and parenting stress. Family Process, 63(4), 2016–2036. 10.1111/famp.13011 38769912

[jcv270006-bib-0009] Defoe, I. N. , Keijsers, L. , Hawk, S. T. , Branje, S. , Dubas, J. S. , Buist, K. , Frijns, T. A. G. , Van Aken, M. , Koot, H. M. , Van Lier, P. A. C. , & Meeus, W. (2013). Siblings versus parents and friends: Longitudinal linkages to adolescent externalizing problems. Journal of Child Psychology and Psychiatry and Allied Disciplines, 54(8), 881–889. 10.1111/jcpp.12049 23398022 PMC3807608

[jcv270006-bib-0010] Eames, C. , Daley, D. , Hutchings, J. , Whitaker, C. J. , Bywater, T. , Jones, K. , & Hughes, J. C. (2010). The impact of group leaders’ behaviour on parents acquisition of key parenting skills during parent training. Behaviour Research and Therapy, 48(12), 1221–1226. 10.1016/j.brat.2010.07.011 20932512

[jcv270006-bib-0011] Eradus, M. , Leijten, P. , Melendez‐Torres, G. J. , Foo, X. Q. , & Oliver, B. R. (2024). Parental differential warmth, hostility, and sibling differences in internalizing and externalizing behavior problems: A meta‐analysis. Journal of Family Psychology, 38(3), 387–399. 10.1037/fam0001194 38271066

[jcv270006-bib-0012] Ferguson, C. J. (2010). Genetic contributions to antisocial personality and behavior: A meta‐analytic review from an evolutionary perspective. Journal of Social Psychology, 150(2), 160–180. 10.1080/00224540903366503 20397592

[jcv270006-bib-0013] Gardner, F. , Burton, J. , & Klimes, I. (2006). Randomised controlled trial of a parenting intervention in the voluntary sector for reducing child conduct problems: Outcomes and mechanisms of change. Journal of Child Psychology and Psychiatry and Allied Disciplines, 47(11), 1123–1132. 10.1111/j.1469-7610.2006.01668.x 17076751

[jcv270006-bib-0014] Hutchings, J. , Gardner, F. , Bywater, T. , Daley, D. , Whitaker, C. , Jones, K. , Eames, C. , & Edwards, R. T. (2007). Parenting intervention in Sure Start services for children at risk of developing conduct disorder: Pragmatic randomised controlled trial. British Medical Journal, 334(7595), 678–682. 10.1136/bmj.39126.620799.55 17350966 PMC1839187

[jcv270006-bib-0015] Leijten, P. , Gardner, F. , Landau, S. , Harris, V. , Mann, J. , Hutchings, J. , Beecham, J. , Bonin, E. M. , & Scott, S. (2018). Research review: Harnessing the power of individual participant data in a meta‐analysis of the benefits and harms of the incredible years parenting program. Journal of Child Psychology and Psychiatry and Allied Disciplines, 59(2), 99–109. 10.1111/jcpp.12781 28696032

[jcv270006-bib-0016] Leijten, P. , Gardner, F. , Melendez‐Torres, G. J. , Weeland, J. , Hutchings, J. , Landau, S. , McGilloway, S. , Overbeek, G. , Van Aar, J. , Menting, A. , Orobio De Castro, B. , Berry, V. , Gaspar, M. F. , Axberg, U. , Morch, W. T. , & Scott, S. (2019). Co‐occurring change in children’s conduct problems and maternal depression: Latent class individual participant data meta‐analysis of the Incredible Years parenting program. Development and Psychopathology, 31(5), 1851–1862. 10.1017/S0954579419001068 31370916

[jcv270006-bib-0017] McGilloway, S. , Mhaille, G. N. , Bywater, T. , Furlong, M. , Leckey, Y. , Kelly, P. , Comiskey, C. , & Donnelly, M. (2012). A parenting intervention for childhood behavioral problems: A randomized controlled trial in disadvantaged community‐based settings. Journal of Consulting and Clinical Psychology, 80(1), 116–127. 10.1037/a0026304 22148879

[jcv270006-bib-0018] McGilloway, S. , NiMhaille, G. , Bywater, T. , Leckey, Y. , Kelly, P. , Furlong, M. , Comiskey, C. , O’Neill, D. , & Donnelly, M. (2014). Reducing child conduct disordered behaviour and improving parent mental health in disadvantaged families: A 12‐month follow‐up and cost analysis of a parenting intervention. European Child and Adolescent Psychiatry, 23(9), 783–794. 10.1007/s00787-013-0499-2 25183424

[jcv270006-bib-0019] McHale, S. M. , Sun, X. , Updegraff, K. A. , & Whiteman, S. D. (2024). Patterns and correlates of changes in sibling intimacy and conflict from middle childhood through young adulthood. Developmental Psychology. 10.1037/dev0001750 PMC11910747 38647474

[jcv270006-bib-0020] Menting, A. T. A. , de Castro, B. O. , Wijngaards‐de Meij, L. D. N. V. , & Matthys, W. (2014). A trial of parent training for mothers being released from incarceration and their children. Journal of Clinical Child and Adolescent Psychology, 43(3), 381–396. 10.1080/15374416.2013.817310 23915290

[jcv270006-bib-0021] Muthén, L. K. , & Muthén, B. O. (2018). Mplus version 8.0. Muthén & Muthén.

[jcv270006-bib-0022] Nylund, K. L. , Asparouhov, T. , & Muthén, B. O. (2007). Deciding on the number of classes in latent class analysis and growth mixture modeling: A Monte Carlo simulation study. Structural Equation Modeling, 14(4), 535–569. 10.1080/10705510701575396

[jcv270006-bib-0023] Oliver, B. R. , & Pike, A. (2018). Mother‐child positivity and negativity: Family‐wide and child‐specific main effects and interactions predict child adjustment. Developmental Psychology, 54(4), 744–756. 10.1037/dev0000467 29239634

[jcv270006-bib-0024] Patterson, G. R. (1982). Coercive family process. Castalia.

[jcv270006-bib-0025] Patterson, G. R. (1984). Siblings: Fellow travelers in coercive family processes. Advances in the Study of Aggression 1, 174–214. Academic Press.

[jcv270006-bib-0026] Pelham, W. E. , Dishion, T. J. , Tein, J. Y. , Shaw, D. S. , & Wilson, M. N. (2017). What doesn’t work for whom? Exploring heterogeneity in responsiveness to the family check‐up in early childhood using a mixture model approach. Prevention Science, 18(8), 911–922. 10.1007/s11121-017-0805-1 28550456 PMC5693624

[jcv270006-bib-0027] Pike, A. , & Oliver, B. R. (2017). Child behavior and sibling relationship quality: A cross‐lagged analysis. Journal of Family Psychology, 31(2), 250–255. 10.1037/fam0000248 27797540 PMC5327865

[jcv270006-bib-0028] Plomin, R. , & Daniels, D. (2011). Why are children in the same family so different from one another? International Journal of Epidemiology, 40(3), 563–582. 10.1093/ije/dyq148 21807642 PMC3147063

[jcv270006-bib-0029] Robinson, E. A. , Eyberg, S. M. , & Ross, A. W. (1980). The standardization of an inventory of child conduct problem behaviors. Journal of Clinical Child Psychology, 9(1), 22–28. 10.1080/15374418009532938

[jcv270006-bib-0030] Sellars, E. , Bowes, L. , Oliver, B. R. , Gardner, F. , Axberg, U. , Berry, V. , Seabra‐Santos, M. J. , Hutchings, J. , McGilloway, S. , Menting, A. T. A. , Overbeek, G. , Scott, S. , & Leijten, P. (2024). Effects of the Incredible Years parenting program on children’s interpersonal conflict: An integrative data analysis. Journal of Family Psychology, 38(6), 847–857. 10.1037/fam0001236 38842871

[jcv270006-bib-0031] Smorti, M. , Inguaggiato, E. , Vezzosi, L. , & Milone, A. (2021). Parenting and sibling relationships in family with disruptive behavior disorders. Are non‐clinical siblings more vulnerable for emotional and behavioral problems? Brain Sciences, 11(10), 1308. 10.3390/brainsci11101308 34679373 PMC8534172

[jcv270006-bib-0032] Tucker, C. J. , & Kazura, K. (2013). Parental responses to school‐aged children’s sibling conflict. Journal of Child and Family Studies, 22(5), 737–745. 10.1007/s10826-013-9741-2

[jcv270006-bib-0033] Van Aar, J. , Leijten, P. , Orobio De Castro, B. , Weeland, J. , Matthys, W. , Chhangur, R. , & Overbeek, G. (2019). Families who benefit and families who do not: Integrating person‐and variable‐centered analyses of parenting intervention responses. Journal of the American Academy of Child & Adolescent Psychiatry, 58(10), 993–1003. 10.1016/j.jaac.2019.02.004 30768388

[jcv270006-bib-0034] Webster‐Stratton, C. (2015). The incredible Years® series: A developmental approach. In M. J. Van Ryzin , K. L. Kumpfer , G. M. Fosco , & M. T. Greenberg (Eds.), Family‐based prevention programs for children and adolescents: Theory, research, and large‐scale dissemination (pp. 42–67). Psychology Press.

[jcv270006-bib-0035] Weeland, J. , Helmerhorst, K. O. W. , & Lucassen, N. (2021). Understanding differential effectiveness of behavioral parent training from a family systems perspective: Families are greater than ‘Some of Their Parts’. Journal of Family Theory and Review, 13(1), 34–57. 10.1111/jftr.12408

