# Supplementary material for: Effects of the Incredible Years parenting program on sibling conduct problems: A latent transition analysis
Source: JCPP Adv. 2025 Apr 16;5(4):e70006. doi: 10.1002/jcv2.70006 (PMC12698276; doi:10.1002/jcv2.70006)
Supplement: Supplementary file 1 — Tables S1–S5 [file JCV2-5-e70006-s001.docx]

# Supporting Information

**
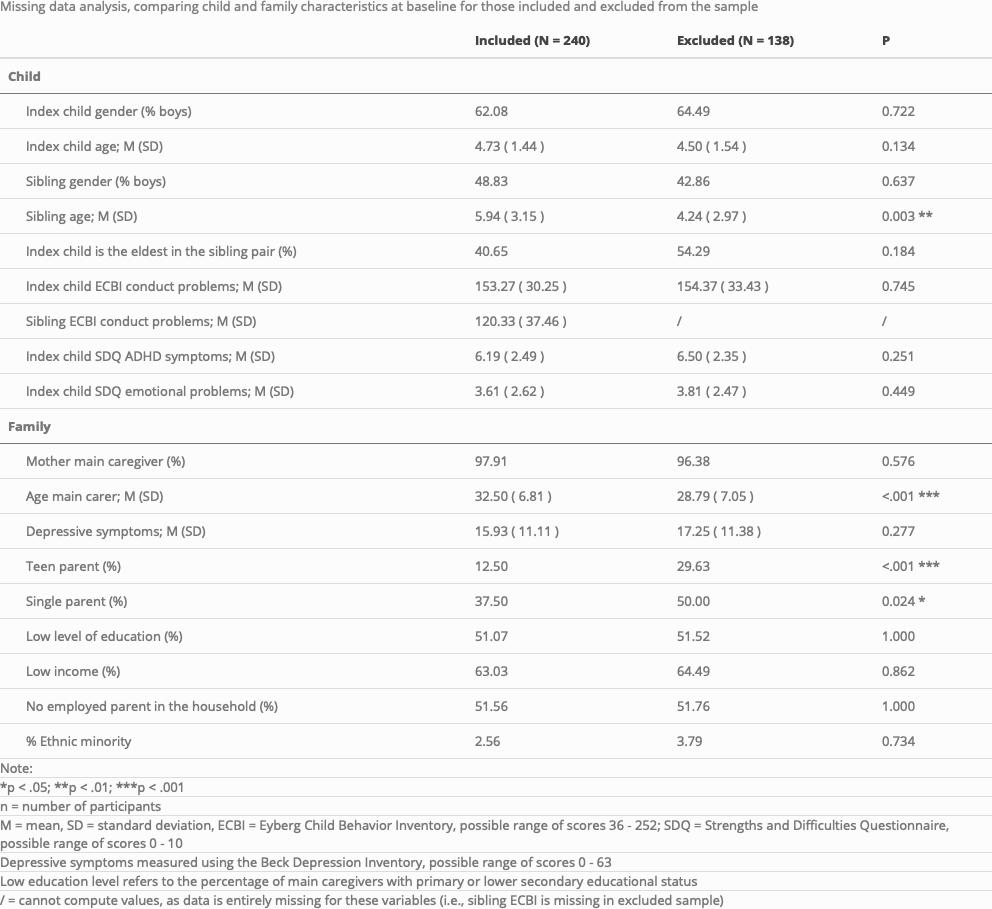
Table S1.** Missing data analysis, comparing child and family characteristics at baseline for those included and excluded from the sample.

**Table S2.** Overview of child and family characteristics at baseline.


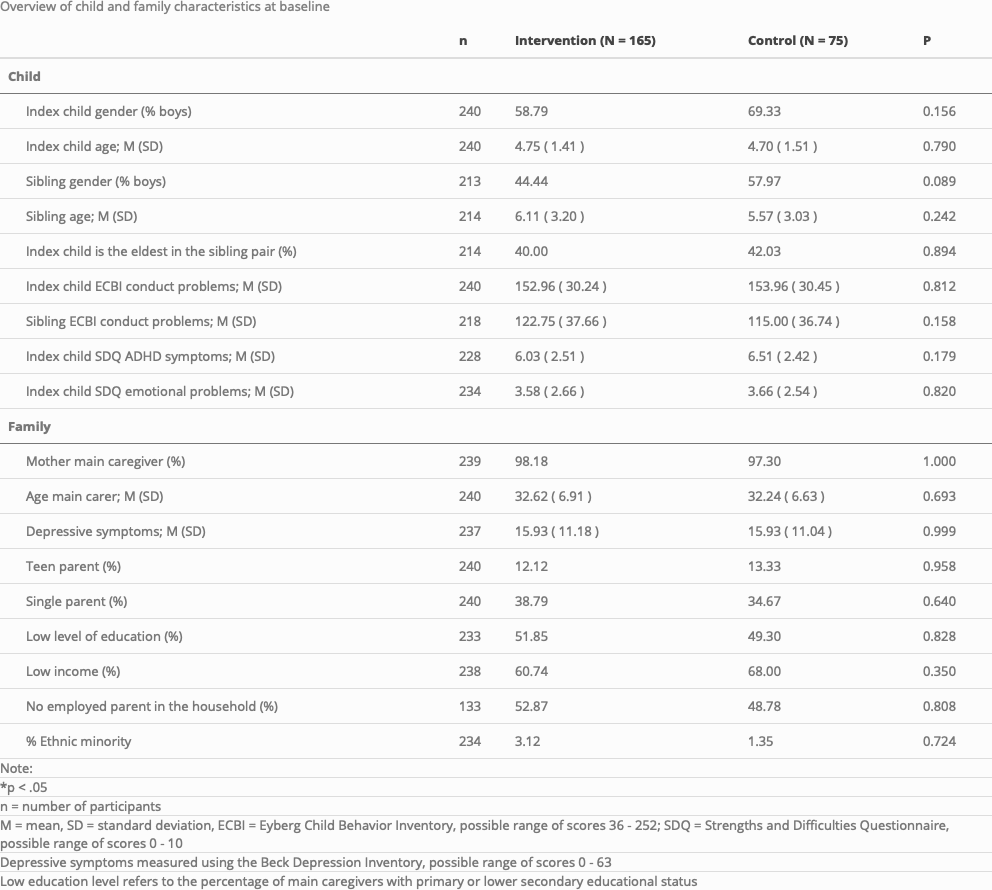


**
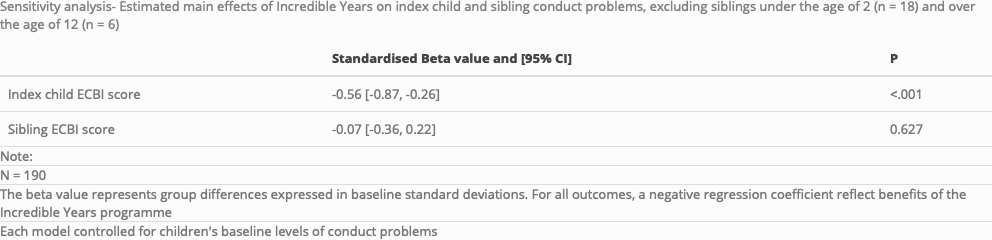
Table S3**. Sensitivity analysis- Estimated main effects of Incredible Years on index child and sibling conduct problems, excluding siblings under the age of 2 (n = 18) and over the age of 12 (n = 6).

**Table S4.** Sibling dyad characteristics according to transition class.


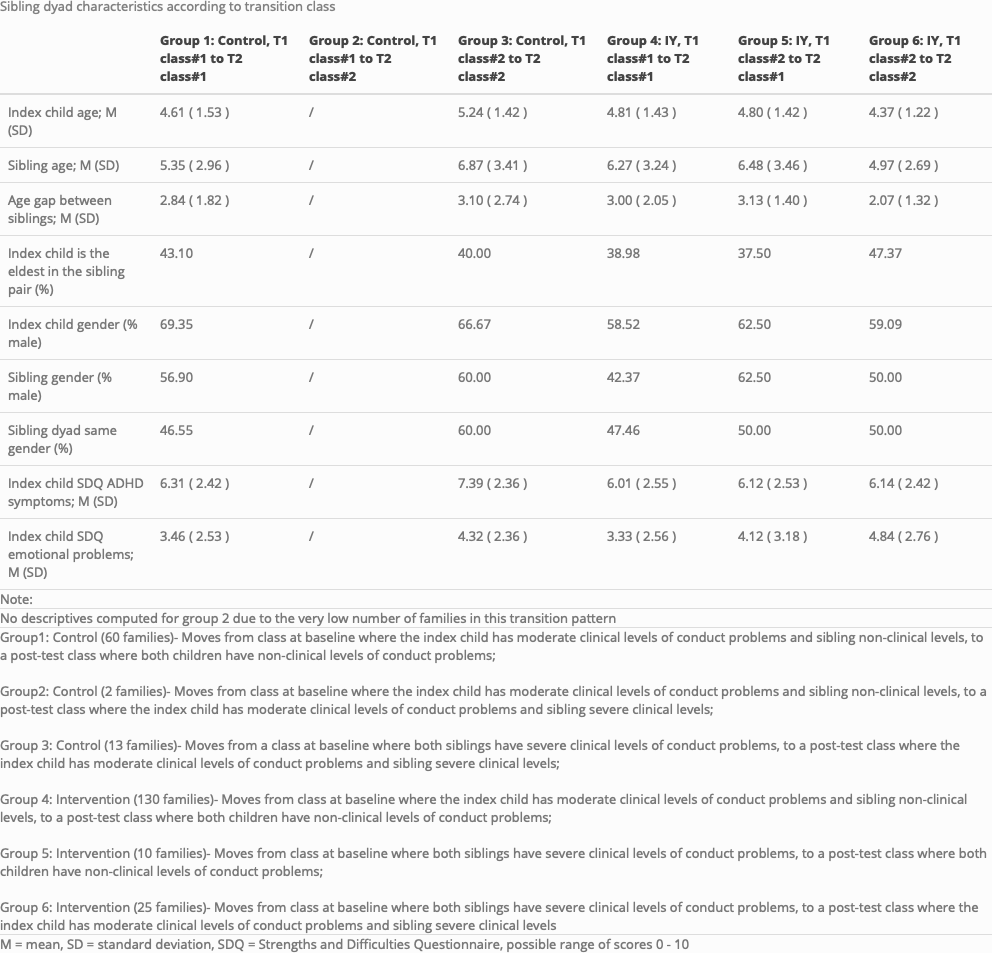


**
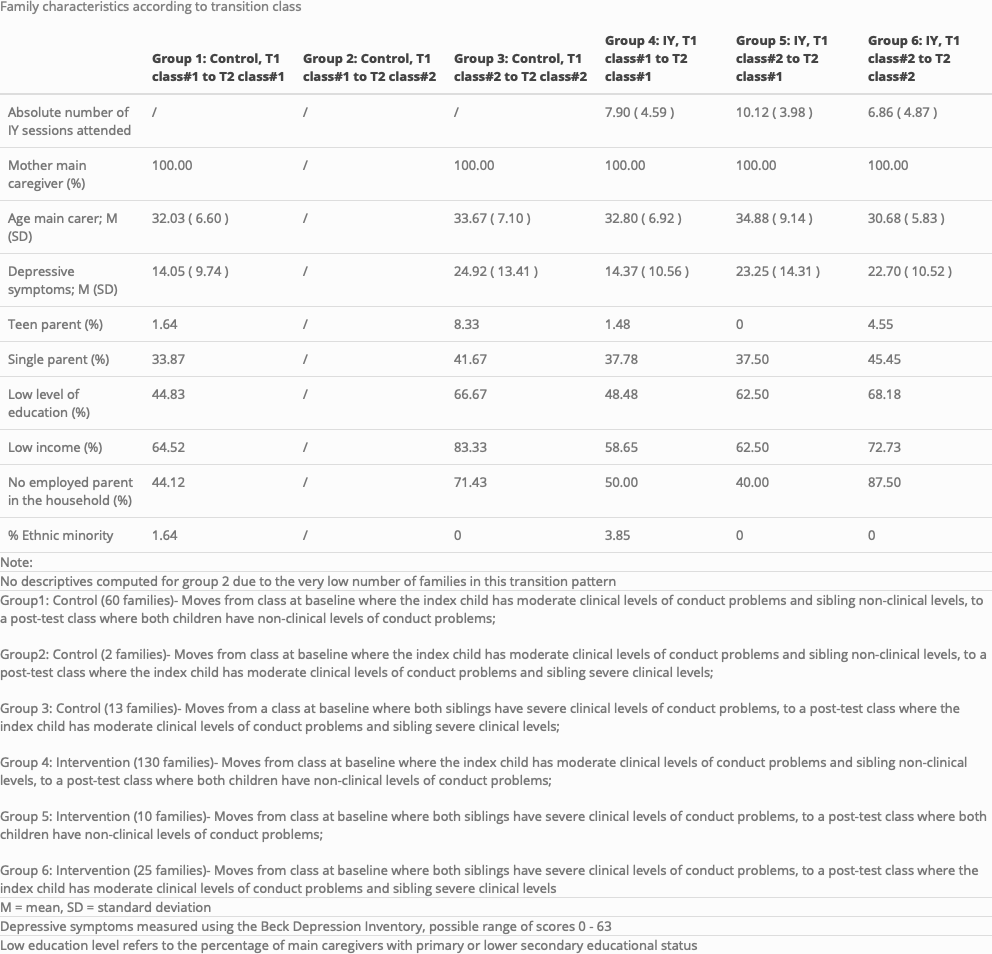
Table S5.** Family characteristics according to transition class.
